# Crystal structure of a novel two domain GH78 family α-rhamnosidase from *Klebsiella oxytoca* with rhamnose bound

**DOI:** 10.1002/prot.24807

**Published:** 2015-08-06

**Authors:** Ellis C O’Neill, Clare E M Stevenson, Michael J Paterson, Martin Rejzek, Anne-Laure Chauvin, David M Lawson, Robert A Field

**Affiliations:** 1 Department of Biological Chemistry, John Innes Centre, Norwich Research ParkNorwich, Nr4 7UH, United Kingdom; 2 Laboratorio Nacional De Genómica Para La Biodiversidad (Langebio), CINVESTAV-IPNIrapuato, Cp36821, México

**Keywords:** α-l-rhamnosidase, glycosyl hydrolase family 78, enzyme structure, flavonoid, rutin, E.C. 3.2.1.40

## Abstract

The crystal structure of the GH78 family α-rhamnosidase from *Klebsiella oxytoca* (KoRha) has been determined at 2.7 Å resolution with rhamnose bound in the active site of the catalytic domain. Curiously, the putative catalytic acid, Asp 222, is preceded by an unusual non-proline *cis*-peptide bond which helps to project the carboxyl group into the active centre. This KoRha homodimeric structure is significantly smaller than those of the other previously determined GH78 structures. Nevertheless, the enzyme displays α-rhamnosidase activity when assayed *in vitro*, suggesting that the additional structural domains found in the related enzymes are dispensible for function. Proteins 2015; 83:1742–1749. © 2015 The Authors. Proteins: Structure, Function, and Bioinformatics Published by Wiley Periodicals, Inc.

## INTRODUCTION

α-l-rhamnosidases (E.C. 3.2.1.40) are found widely distributed in nature and have been reported in animals, plants, yeast, fungi and bacteria, where they are responsible for the cleavage of α-l-rhamnose from a wide range of compounds.^1^ α-l-rhamnose is found in plants and bacteria as components of polysaccharides, such as pectins,^2^ and the O antigen polysaccharides, responsible for determining the antigenicity of pathogenic bacteria^3^; it is also found in rhamnolipids^4^ and it is attached to small molecule natural products, such as rutin. There is industrial interest in α-rhamnosidases for use in the debittering of citrus juices and for the release of flavonoids from rhamnosylated precursors; in wine production they play a role in the hydrolysis of glycosylated terpene aroma compounds.^5^ In the context of expanding and diversifying the suite of enzymes available to us for carbohydrate biotransformations,^6^–^13^ we were drawn to consider α-rhamnosidases.

In the CAZy database,^14^ rhamnosidases are currently classified into GH28 pectin hydrolases, GH78 containing exclusively rhamnosidases, and GH106 containing a single enzyme from *Sphingomonas*.^15^ The family GH78 α-l-rhamnosidases catalyse the hydrolysis of α-l-rhamnosyl-linkages through an acid-base catalysed single displacement (inverting) mechanism.^16^,^17^ To date, three structures have been determined from the GH78 rhamnosidase family (Supporting Information Fig. S1 and Table S1). The first crystal structure determined was for α-l-rhamnosidase B from *Bacillus sp*. GL1 (BsRhaB; PDB code 2OKX).^18^ This protein is homodimeric, consisting of four β-sandwich domains and a core catalytic (α/α)_6_ barrel. The crystal had been soaked with the product l-rhamnose and additional electron density was observed in a deep cleft of the (α/α)_6_ barrel, although the sugar could not be modelled into this density. More recently, the structure of another α-l-rhamnosidase from *Streptomyces avermitilis* (SaRha78A; PDB code 3W5N) was solved in complex with l-rhamnose. This protein is monomeric and larger, consisting of six domains. The product, l-rhamnose, could be seen bound at the active site in the core catalytic (α/α)_6_ barrel domain, but also bound to a novel calcium-dependent, non-catalytic carbohydrate binding module (CBM67).^19^ The third structure, also a homodimer, determined in a structural genomics project is unpublished, but is a putative α-l-rhamnosidase from *Bacteroides thetaiotaomicron* VP1-5482 (BT1001; PDB code 3CIH). This enzyme is the smallest of the deposited structures, consisting of only four domains. In these three previously determined structures, there is substantial variation in the additional domains appended to the catalytic core. This current work describes an even smaller putative α-l-rhamnosidase that has been identified in *Klebsiella oxytoca* (UniProtKB entry X5FHT8), a Gram negative nitrogen fixing bacterium closely related to the human pathogen *Klebsiella pneumoniae*. The *K. oxytoca* enzyme was expressed in *E. coli* and the protein purified. Having confirmed activity against an aryl rhamnoside and a rhamnose-containing flavanone, the protein was crystallized and its structure determined to reveal a core (α/α)_6_ barrel and just one additional β-sandwich domain.

## METHODS

### Protein expression and purification

The putative α-l-rhamnosidase (EC 3.2.1.40) from *K. oxytoca* KCTC 1686^20^ (KoRha) was identified by homology with the *Pediococccus acidilactici ram* gene.^5^ The synthetic gene for KoRha (accession YP_005019950), synthesized by Genscript with codon optimization for expression in *E. coli*, was cloned using the NdeI and XhoI restriction sites of the pET15b expression vector, inserting an additional N-terminal thrombin cleavable His_6_-tag with sequence MGSSHHHHHHSSGLVPRGSH. This construct was introduced into *E. coli* BL21(DE3) by heat shock-mediated transformation. The protein was expressed by inoculating 1 L of auto induction media containing 100 mg L^−1^ carbenicillin with 10 mL of an overnight culture of these cells, followed by incubation overnight at 22°C with shaking at 200 rpm. The cells were harvested by centrifugation at 4°C and resuspended in 50 mL buffer A (20 m*M* HEPES [2-[4-(2-hydroxyethyl)piperazin-1-yl]ethanesulfonic acid] pH 7.5, 150 m*M* NaCl, and 30 m*M* imidazole) with the addition of a Complete^TM^ EDTA protease inhibitor tablet (Roche), 0.1 mg mL^−1^ lysozyme, and 0.01 mg mL^−1^ DNaseI (Sigma). Cells were lysed by sonication and insoluble material was removed by centrifugation at 4°C. The protein was purified using an ÄKTAxpress (GE Healthcare), which loaded the supernatant onto a 5 mL His-Trap chelating HP column. The column was then washed with buffer A, and bound protein was eluted in one step with buffer A containing 500 m*M* imidazole. The eluted protein was then injected directly onto a Superdex 75 26/60 gel filtration column that had been pre-equilibrated with buffer B (20 m*M* HEPES pH 7.5 and 150 m*M* NaCl). Fractions containing KoRha (as judged by SDS-PAGE [sodium dodecyl sulfate polyacrylamide gel electrophoresis]) were collected, pooled, and concentrated to 2.1 mg mL^−1^ using an Amicon Ultra 30 kDa MW cut off concentrator. The protein yield was approximately 8 mg L^−1^ of culture and the purity judged to be 95% by SDS-PAGE. The His tag was not cleaved and the protein was stored in aliquots at −80°C.

Prior to crystallization, dynamic light scattering was used to monitor the solution properties of the purified sample with a DynaPro-Titan molecular-sizing instrument at 20°C (Wyatt Technology). The protein sample gave a hydrodynamic radius of 4.8 nm, which corresponds to a molecular size of 117 kDa, approximately twice the calculated mass of the monomer (61.6 kDa).

### Crystallization and data collection

KoRha was crystallized at 10 mg mL^−1^ in buffer B with the addition of 100 m*M* rhamnose. All crystallizations were performed at a constant temperature of 20°C. Screening was carried out with an OryxNano robot (Douglas instruments Ltd) in 96-well sitting drop MRC plates (Molecular Dimensions) using a variety of commercial screens (Molecular Dimensions and Qiagen). Promising conditions were optimized manually in a 24-well hanging drop vapor diffusion format using VDX plates (Molecular Dimensions) with a reservoir volume of 1 mL and drops consisting of 1 µL protein and 1 µL well solution. Protein crystals appeared after 7 days from conditions containing 10% (wt/vol) PEG 3350 and 200 m*M* MgSO_4_. Crystals were manipulated using LithoLoops (Molecular Dimensions) and transferred to a cryoprotectant solution containing the crystallization condition with the addition of 20% (vol/vol) ethylene glycol. Crystals were flash-cooled in liquid nitrogen and stored in Unipuck cassettes (MiTeGen) prior to transport to the synchrotron. For experimental phasing a single crystal was soaked for approximately 1 min in the cryoprotectant solution containing 0.5*M* NaBr.

Crystals were subsequently transferred robotically to the goniostat on the beamline at the Diamond Light Source (Oxfordshire, UK) and maintained at −173°C with a Cryojet cryocooler (Oxford Instruments). Native diffraction data were recorded on beamline I24 (wavelength = 1.068 Å) using a Pilatus 6M detector (Dectris). A highly redundant derivative data set was collected on a bromide-soaked crystal on beamline I04-1 (wavelength 0.920 Å) using a Pilatus 2M detector (Dectris). All X-ray data were processed using Xia2^21^ and the resultant data collection statistics are summarized in Table1. The crystals belonged to space group *P4_1_2_1_2* with approximate cell parameters of *a* = *b* = 148, *c* = 202 Å.

**Table 1:** X-Ray Data Collection and Refinement Statistics for α-l-Rhamnosidase From *Klebsiella oxytoca* (KoRha)

	Native	Bromide derivative
Data collection		
Beamline	I24, Diamond Light Source, UK	I04-1, Diamond Light Source, UK
Wavelength (Å)	1.068	0.920
Detector	Pilatus 6M	Pilatus 2M
Resolution range (Å)	63.07–2.70 (2.77–2.70)	95.58–2.70 (2.77–2.70)
Space Group	*P4_1_2_1_2*	*P4_1_2_1_2*
Cell parameters (Å)	*a* = *b* = 148.43, *c* = 202.16	*a* = *b* = 147.40, *c* = 201.56
Total no. of measured intensities	300,908 (22,055)	4,618,293 (311,023)
Unique reflections	61,251 (4450)	61,461 (4462)
Multiplicity	4.9 (5.0)	75.1 (69.7)
Mean *I*/σ(*I*)	9.7 (1.4)	34.4 (3.8)
Completeness (%)	98.5 (99.0)	99.8 (99.8)
*R*_merge_^a^	0.111 (0.970)	0.174 (1.653)
*R*_meas_^b^	0.137 (1.199)	0.176 (1.677)
*CC*_½_[Table-fn tf1-3]	0.995 (0.625)	1.000 (0.897)
Wilson *B* value (Å^2^)	41.8	48.8
Refinement		
Resolution range (Å)	63.07–2.70 (2.77–2.70)	—
Reflections: working/free^c^	58,166/3084	—
*R*_work_/*R*_free_^d^	0.182/0.202 (0.361/0.419)	—
Ramachandran plot: favored/allowed/disallowed^f^ (%)	97.3/100.0/0.0	—
R.m.s. bond distance deviation (Å)	0.007	—
R.m.s. bond angle deviation (°)	1.134	—
No. of protein residues: chain A/chain B	512 (11–523)/512 (11–523)	—
No. of water molecules/sulfate ions/rhamnose sugars	45/4/2	—
Mean *B* factors: protein/water/sulfate/rhamnose/overall (Å^2^)	57.4/44.7/101.6/43.8/57.3	—
PDB accession code	4XHC	—

Values in parentheses are for the outer resolution shell.

a *R*_merge_ = ∑*_hkl_* ∑*_i_* |*I_i_*(*hkl*) − ⟨*I*(*hkl*)⟩|/∑*_hkl_* ∑*_i_I_i_*(*hkl*).

b *R*_meas_ = ∑*_hkl_* [*N*/(*N* − 1)]^1/2^ × ∑*_i_* |*I_i_(hkl)* − ⟨*I(hkl)*⟩|/∑*_hkl_* ∑*_i_I_i_(hkl)*, where *I_i_(hkl)* is the *i*th observation of reflection *hkl*, ⟨*I(hkl)*⟩ is the weighted average intensity for all observations *i* of reflection *hkl* and *N* is the number of observations of reflection *hkl*.

c CC_½_ is the correlation coefficient between symmetry equivalent intensities from random halves of the dataset.

d The data set was split into “working” and “free” sets consisting of 95 and 5% of the data respectively. The free set was not used for refinement.

e The *R*-factors *R*_work_ and *R*_free_ are calculated as follows: *R* = ∑(| *F*_obs_ − *F*_calc_ |)/∑| *F*_obs_ | × 100, where *F*_obs_ and *F*_calc_ are the observed and calculated structure factor amplitudes, respectively.

f As calculated using MolProbity.^27^

### Structure solution

The structure was solved using the data from the crystal soaked in bromide using the single wavelength anomalous diffraction approach. HKL2MAP^22^ was used to run the SHELX suite of programs. SHELXC indicated that there was a strong anomalous signal and SHELXD was successful in finding 43 bromide sites (with an occupancy greater than 0.3) using data in the resolution range 40–4 Å. Density modification and twofold averaging was carried out using PARROT^23^ and a good quality experimentally phased electron density map was obtained. BUCCANEER^24^ was able to successfully build into this map assigning 96% of the sequence to two chains with *R*_work_ and *R*_free_ values of 0.256 and 0.298, respectively, and a figure-of-merit (FOM) of 0.77 at 2.7 Å resolution. Several iterations of model building with COOT^25^ and restrained refinement with REFMAC5^26^ were carried out against the native data to 2.7 Å. The final model consisted of 1024 residues (in two polypeptide chains each containing residues 11–523 of the expected wild-type sequence), 45 water molecules, four sulfate ions, and two rhamnose molecules. The final *R*_work_ and *R*_free_ values were 0.182 and 0.202, respectively, with a FOM of 0.83 to 2.7 Å resolution (refinement statistics are summarized in Table1). MolProbity^27^ was used to validate the model before deposition in the PDB with accession code 4XHC. All structural figures were prepared using CCP4MG.^28^

### Activity assay and pH optimum

The activity of KoRha was measured using 4-nitrophenyl-α-l-rhamnoside (pNP-Rha).^29^ To determine the optimum pH for this activity pNP-Rha (5 m*M*) was dissolved in the assay buffer (20 m*M* acetate, 20 m*M* MES, 20 m*M* Tris), at a range of pH values (4–10), containing 5% (vol/vol) dimethyl sulfoxide (DMSO) and KoRha was added to a final concentration of 21 µg mL^−1^. After 1 h at 22°C, the reaction was terminated by the addition of an equal volume of 2*M* aqueous Na_2_CO_3_ solution and the absorbance was measured at 415 nm. In order to determine the activity of KoRha, pNP-Rha was dissolved at a range of concentrations (0–10 m*M*) in 10% (vol/vol) DMSO, and the assay was performed in assay buffer (20 m*M* MES pH 5.0), using GraFit (Erithacus Software) to calculate kinetic parameters. The absorbance at 415 nm was measured relative to standard amounts of pNP in 1% (vol/vol) DMSO and 20 m*M* MES pH 5.0.

The ability of KoRha to cleave α-l-rhamnose from rutin was monitored using thin layer chromatography (TLC). Rutin (10 m*M*) was incubated in the presence of KoRha (100 µg mL^−1^) in buffer (MES 20 m*M*, pH 5.0) containing 20% (vol/vol) methanol for 16 h at 22°C. The reaction products were separated by TLC (Merck silica gel 60 F_254_) using a mobile phase of 100:11:11:25 (EtOAc:AcOH:HCOOH:H_2_O). Absorbent components were visualized by UV (254 nm) and by dipping in orcinol and charring.

## RESULTS AND DISCUSSION

### KoRha structure

The crystal structure of KoRha with rhamnose bound was determined to 2.7 Å resolution. The final model consisted of two KoRha subunits related by a non-crystallographic twofold axis (giving a corresponding solvent content of 73%) in the asymmetric unit, with each monomer containing a bound rhamnose. Dynamic light scattering had suggested that KoRha was a homodimer in solution and the structure of KoRha confirmed this, giving a dimer interface of 1389.9 Å^2^ (as calculated using the PISA server (http://www.ebi.ac.uk/pdbe/pisa/).

**Figure 1:**
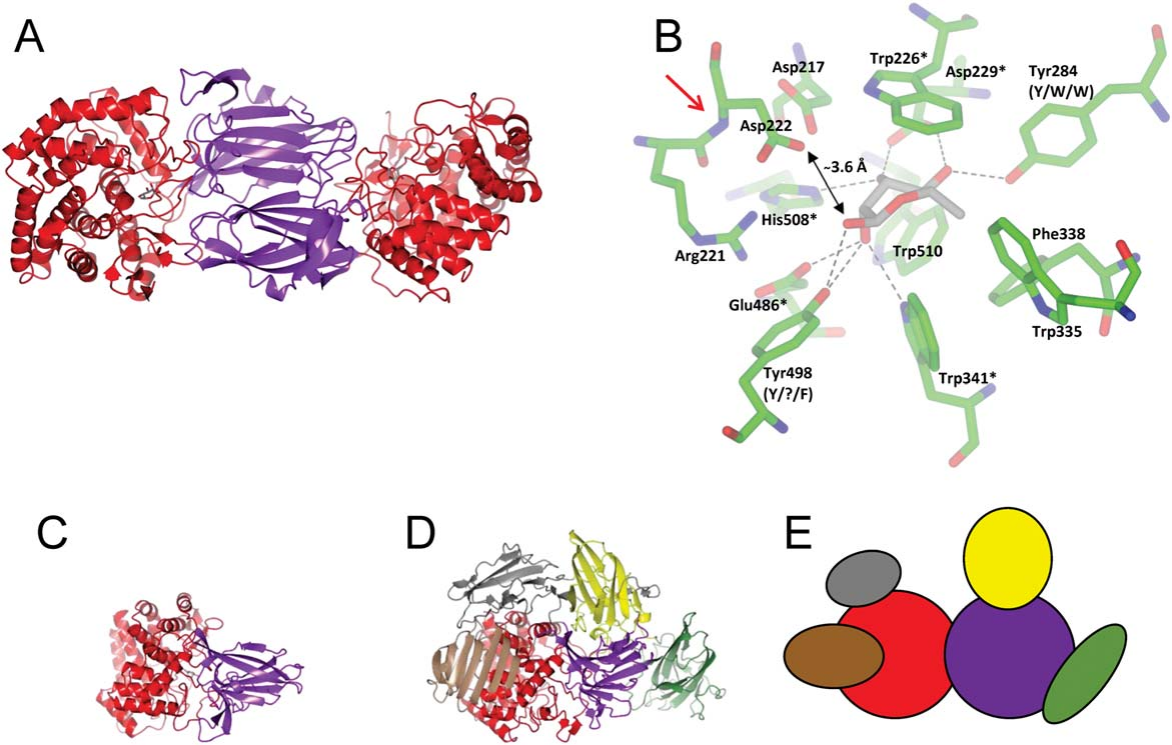
X-ray crystal structure of the α-l-rhamnosidase from *K. oxytoca* (KoRha). A: Cartoon representation of the KoRha dimer. Each monomer consists of two domains: domain A is shown in red and domain B in purple. Rhamnose is bound to each monomer and is shown in gray and red. B: Rhamnose binding site. The rhamnose is shown in light gray and all the residues within 4 Å of the rhamnose are shown in green and are labeled. Residues that hydrogen bond to the rhamnose are marked with an asterix if they are conserved in the other three deposited α-l-rhamnosidase structures. The conserved Trp 226 is also labeled with an asterix. Where the residues that hydrogen bond to the rhamnose are not conserved, the equivalent residues are labeled with the α-l-rhamnosidase from *B. thetaiotaomicron* VP1-5482 (BT1001) first, the α-l-rhamnosidase B from *Bacillus sp*. GL1 (BsRhaB) second, and the α-l-rhamnosidase from *S. avermitilis* (SaRha78a) third. For Tyr 498 there is no equivalent amino acid in BsRhaB, so this has been indicated with a question mark. A double headed arrow shows the distance from Asp 222 to the O1 of the rhamnose. A red arrow indicates the position of the non proline *cis*-peptide bond. C: Cartoon representation of the KoRha monomer with domain A colored red and domain B purple. D: Cartoon representation of SaRha78a, the largest of the deposited structures. This is in the same orientation as KoRha and the equivalent domains are colored the same. E: Diagram of the SaRha78a structure with each domain depicted with a different color. The red and purple domains are common to all the α-l-rhamnosidase structures.

Each monomer of KoRha is composed of two domains. Domain A, the catalytic domain, is mainly α-helical, consisting of residues 11–30 and 180–523, and contains the bound rhamnose. Domain B, the dimerization domain, is a β-sandwich domain consisting of residues 31–179. Figure 1(A) illustrates the KoRha homodimer with domain A colored red and domain B purple.

### Comparison with other GH78 α-rhamnosidase structures

The KoRha subunit is significantly smaller than for the other three published structures, which all contain additional domains (Supporting Information Fig. S1 and Table S1). The largest structure is SaRha78a, which contains six domains, compared to the two domains present in KoRha. Comparison between the two structures shows that the two domains of KoRha match two of the six domains in SaRha78a [Fig. 1(C–E)]. One of the extra domains present in SaRha78a (domain D—green) has been characterized as a non-catalytic carbohydrate-binding module, which has been shown to increase enzyme activity against insoluble substrates.^19^ The function of the other domains remains to be elucidated.

**Figure 2:**
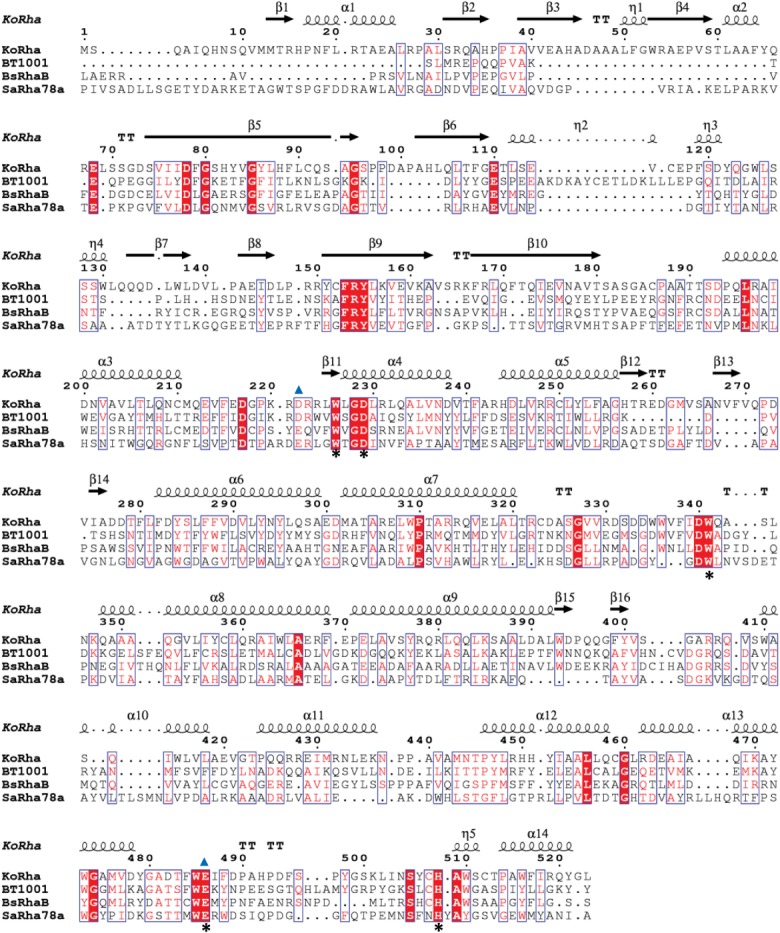
Structure-based multiple sequence alignment of the α-l-rhamnosidase from *K. oxytoca* (KoRha) with α-l-rhamnosidase from *B. thetaiotaomicron* VP1-5482 (BT1001, PDB code: 2CIH), α-l-rhamnosidase B from *Bacillus sp*. GL1 (BsRhaB, PDB code: 2OKX), and α-l-rhamnosidase from *S. avermitilis* (SaRha78a, PDB code: 3W5N). The initial alignment was generated using T-Coffee (http://www.ebi.ac.uk/Tools/msa/tcoffee/), manually adjusted, and then displayed using ESPript3 (http://espript.ibcp.fr/ESPript/ESPript/). Strictly conserved residues are highlighted with red shaded boxes, and moderately conserved residues are boxed. Secondary structural elements for KoRha are shown above the alignment, where α = α helix, β = β strand, η = 3_10_ helix. Strictly conserved residues involved in rhamnose binding are marked with an asterisk. The proposed catalytic residues are marked with a triangle. [Color figure can be viewed in the online issue, which is available at wileyonlinelibrary.com.]

Sequence identities between the GH78 proteins are low, with KoRha having 27% identity over 383 amino acids to BsRhaB, 25% identity to BT1001 over 345 amino acids, and 20% identity to SaRha78a over 340 amino acids. However, the two domains present in the KoRha structure overlay well with the equivalent domains in the other published structures (Supporting Information Fig. S1). When compared using Secondary Structure Matching,^30^ KoRha aligns closest with BsRhaB with a root mean square deviation (rmsd) of 1.95 Å over 428 residues. The alignment with BT1001 has a rmsd of 2.42 Å over 411 residues, and with SaRhaB, a rmsd of 2.66 Å over 386 residues. The domain information for all four structures can be found in the Supporting Information Table S1, and this information was used to truncate the other sequences, such that they contained only the two domains present in KoRha. These truncated sequences were then used to produce the structure-based sequence alignment shown in Figure 2. For SaRhA78A, this corresponds to Domains A and F (residues 427–931); for BsRhaB Domains A and D2 (residues 406–875); and BT1001 (residues 175–636). Like KoRha, BsRhaB and BT1001 are homodimeric, but the quaternary structure is not conserved between the three enzymes.

### Rhamnose binding site

The SaRha78A structure has previously been solved with and without rhamnose bound, and there was little observed effect of ligand binding on the structure.^19^ The structure of KoRha crystallized in the presence of rhamnose shows the sugar bound to Domain A in each monomer. The rhamnose was modeled in a distorted chair conformation. It is bound at the bottom of a deep pocket, in a narrow inner chamber [Fig. 1(B) and Supporting Information Figs. S2 and S3], where the pyranose ring is sandwiched between Trp 341 and Trp 226 (which are structurally conserved in the other three determined structures) and flanked by other hydrophobic residues, including Trp 335, Phe 338, and Trp 510. The rhamnose makes eight direct hydrogen bonds with KoRha: the O-1 atom with Tyr 498, O-2 with the putative catalytic Glu 486, and Trp 341 (both conserved in the other three structures) and also with Tyr 498, which is only conserved in BT1001. In addition, the O-3 atom makes two hydrogen bonds with Asp 229 and His 508 (both also conserved). O-4 also hydrogen bonds to the conserved Asp 229 and additionally to Tyr 284, which is conserved in BT1001, but replaced by a Trp in SaRha78A and BsRhaB.

Asp 222 most likely serves as the catalytic acid, responsible for protonating the oxygen of the scissile glycosidic bond of the rhamnoside substrate. It is notable that this residue is preceded by an unusual non-proline *cis*-peptide bond, which helps to project the carboxyl group into the active center. In contrast, SaRha78A and BsRhaB have Glu residues at this position, and the preceding bonds are *trans*; presumably the longer side-chain makes a *cis*-peptide unnecessary in these enzymes. By comparison, the carboxyl group of Asp 222 is ∼3.6 Å from O-1 of the bound rhamnose in KoRha, and the carboxyl group of Glu 636 is ∼3.5 Å from O-1 of the bound rhamnose in SaRha78A. However, like KoRha, the putative catalytic acid in BT1001 is also an Asp, but the preceding peptide bond has been modeled as *trans*. Closer inspection of the electron density for the latter indicates that this should be remodeled as *cis*. When this peptide bond was remodeled and refined in the *cis* configuration, there was good agreement with the resulting electron density (Supporting information Fig. S4).

The single displacement inverting mechanism proposed for GH78 α-l-rhamnosidases invokes the participation of a nucleophilic water molecule that, in KoRha, would most likely be activated by Glu 486. However, our structure reveals no evidence for a water molecule in the vicinity of the β-anomeric position and within hydrogen bonding distance of Glu 486. One could argue that a “catalytic” water was not visible due to the limited resolution of the X-ray data. However, the disposition of the protein side chains around the active site, especially Tyr 498, does not allow space for an appropriately placed water molecule. Thus, this structure may not fully mimic a catalytically competent complex.

The O-1 atom of rhamnose faces a solvent exposed outer chamber, which presumably corresponds to the aglycone binding site. This is formed by residues from both subunits and is more spacious than the inner chamber, perhaps indicating flexibility for the rhamnoside substrate (Supporting Information Fig. S2).

### Activity of KoRha

The α-rhamnosidase activity of KoRha was assayed using 4-nitrophenol release (pNP) from pNP-Rha (Supporting Information Fig. S5). The pH optimum of KoRha was found to be approximately 5.0 in a mixed buffer system. At this pH, *V*_max_ was found to be 0.95 ± 0.003 μmol mg^−1^ min^−1^, which is equivalent to a turnover of 0.98 s^−1^, and the *K*_M_ for pNP-Rha is 0.21 ± 0.02 m*M*. The activity assay data are shown in Supporting Information Figure S5.

The ability of this enzyme to hydrolyze rhamnose from rutin, a rhamnosylated flavonol, was determined using TLC. After incubation in the presence of the KoRha, two new spots appeared, consistent with the production of rhamnose and arhamnosyl rutin (quercetin-3-β-d-glucoside) (Supporting Information Fig. S6). These properties are consistent with other enzymes of this type and indicate KoRha may prove to be useful for the general hydrolysis of rhamnosides.

## CONCLUSIONS

We have determined the crystal structure of KoRha, a putative α-l-rhamnosidase from *K. oxytoca*, to 2.7 Å resolution with rhamnose bound. The structure reveals an elongated homodimer, with each protomer comprised of two domains. One domain is largely responsible for forming the dimer interface, whilst the other is the catalytic domain and the site of rhamnose binding. The ligand is buried in a narrow chamber at the bottom of a deep pocket, making numerous interactions with the protein. The reducing end of the rhamnose faces a more spacious chamber at the mouth of the pocket, which presumably represents a relatively non-specific aglycone binding site. These observations are consistent with KoRha being an exclusively exo-acting enzyme, removing rhamnose from the non-reducing end of substrates. The putative catalytic carboxylic acid residues flank the C-1 hydroxyl of the rhamnose, and the carboxyl group of the presumed proton donor is correctly presented to the substrate through the incorporation of a non-proline *cis*-peptide bond between it and the preceding residue. KoRha is significantly smaller than the other known rhamnosidase structures, yet it is catalytically active, being able to hydrolyze rhamnose from rutin *in vitro*. Given its comparative compactness and its likely substrate promiscuity, KoRha offers potential as a general α-rhamnosidase with scope for industrial applications.
