# Supplementary material for: Crystal structure of a novel two domain GH78 family α-rhamnosidase from Klebsiella oxytoca with rhamnose bound
Source: Proteins. 2015 Aug 6;83(9):1742–9. doi: 10.1002/prot.24807 (PMC4690510; doi:10.1002/prot.24807)
Supplement: Supplementary file 1 [file prot0083-1742-sd1.pdf]

α-L-rhamnoside	SaRha78A	BsRhaB	BT1001	KoRha
Organism	*Streptomyces avermitilis*	*Bacillus sp.* GL1	*Bacteroides thetaiotaomicron*	*Klebsiella oxytoca*
PDB code	3W5N	2OKX	3CIH	4XHC
Rmsd after superposition with KoRha (aligned residues)	2.66 Å (386)	1.95 Å (428)	2.42 Å (411)	-
Molecular weight of monomer (kDa)	113.4	106.1	85.1	61.6
Number of domains	6	5	4	2
Colour of domain	Domain definition<br>Residues included			
Grey	Domain N<br>3-114	-	-	-
Yellow	Domain E<br>115-132<br>298-426<br>444-448	Domain N<br>3-141<br>157-192	18-174	-
Green	Domain D<br>133-297	Domain D1<br>219-403	-	-
Purple	Domain F<br>427-443<br>479-596	Domain D2<br>153-155<br>210-215<br>406-528	175-296	Domain B<br>31-179
Red	Domain A<br>606-931	Domain A<br>543-875	297-636	Domain A<br>11-30<br>180-523
Brown	Domain C<br>597-605<br>932-1030	Domain C<br>879-954	637-731	-
Biological Assembly	monomer	dimer	dimer	dimer

Supporting Information Table S1

Information and domain definitions for KoRha and the deposited GH78 α-L-rhamnosidase structures.

Domain definitions had not been assigned for the BT1001 structure. The domains were identified by overlaying this deposited structure with the other structures and manually assigning the domains.

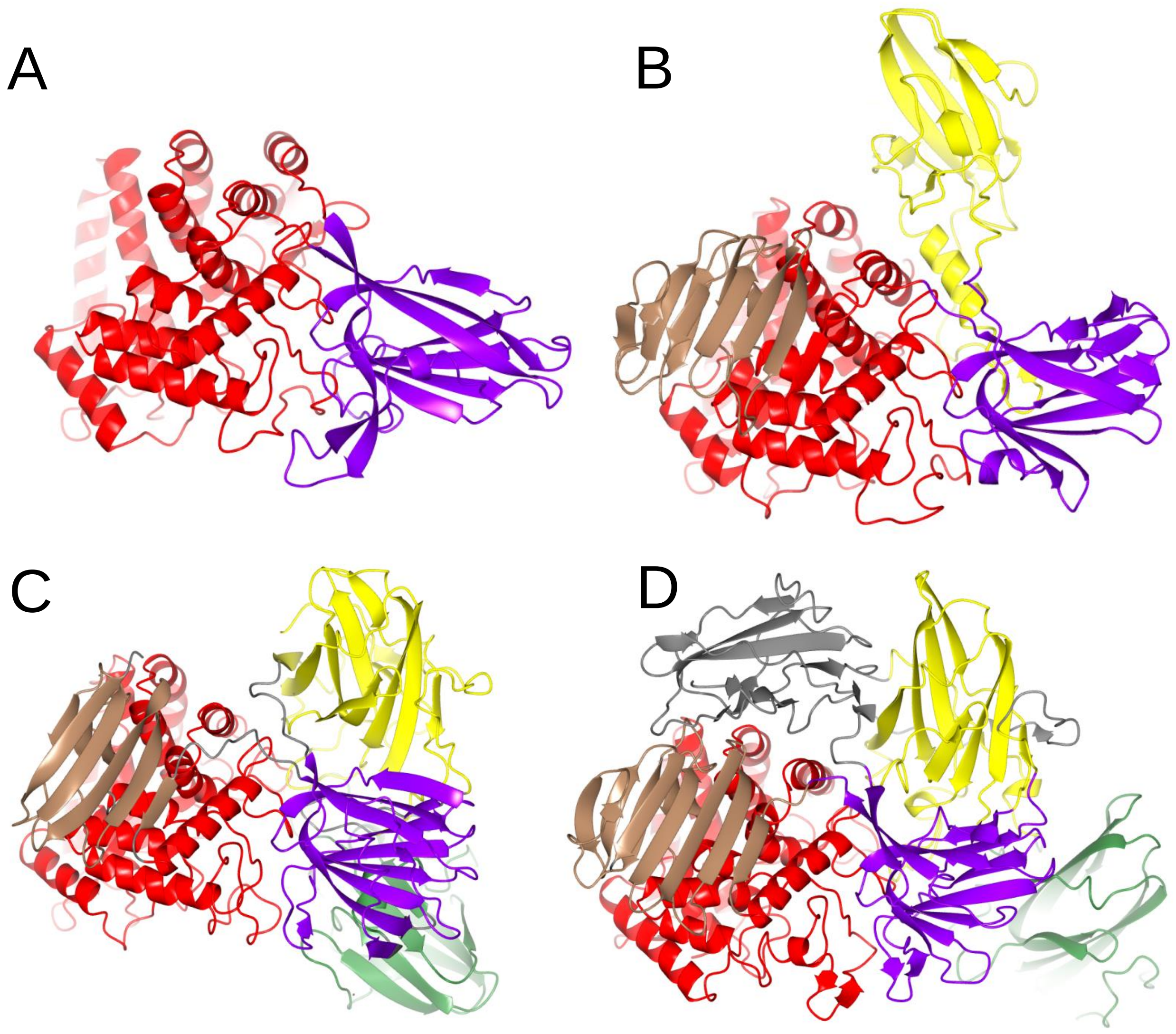

Supporting Information Fig. S1

Cartoon representation of all the α-L-rhamnosidase structures shown in the same orientation. A: KoRha structure consists of two domains. Domain A is coloured red and domain B purple. These domains are present in the other three structures. B: α-L-rhamnosidase from *Bacteroides thetaiotaomicron* VP1-5482 (BT1001) consists of four domains. C: α-L-Rhamnosidase B from *Bacillus sp*. GL1 (BsRhaB) consists of five domains. D: α-L-Rhamnosidase from *Streptomyces avermitilis* (SaRha78a) has six domains.

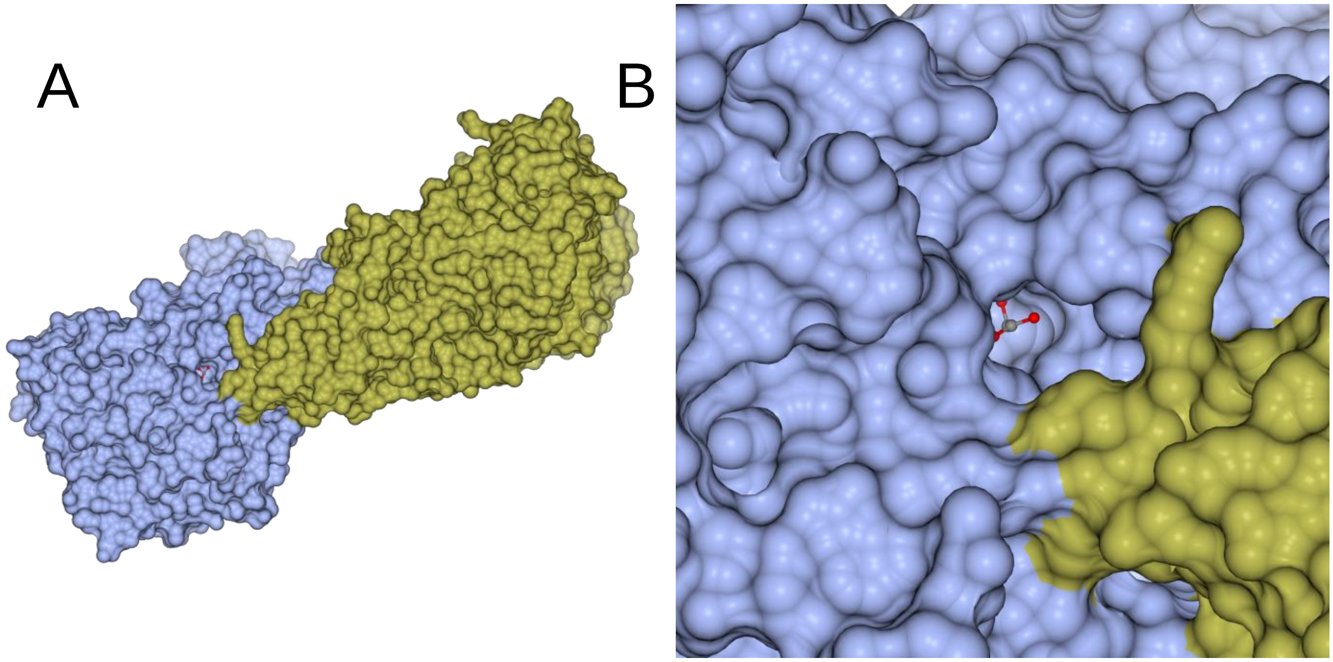

Supporting Information Fig. S2

A: Space filling representation of the KoRha dimer. One monomer is coloured blue and the other monomer olive green. B: Close up of the binding pocket. The O-1 atom of the rhamnose can be seen projecting out of the binding pocket.

A

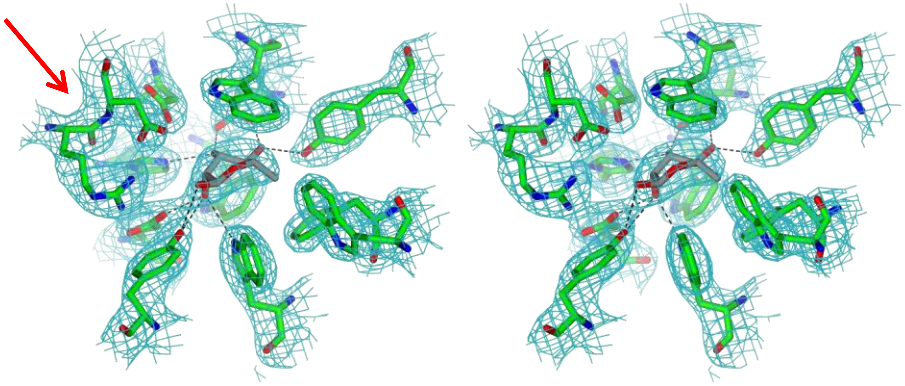

B

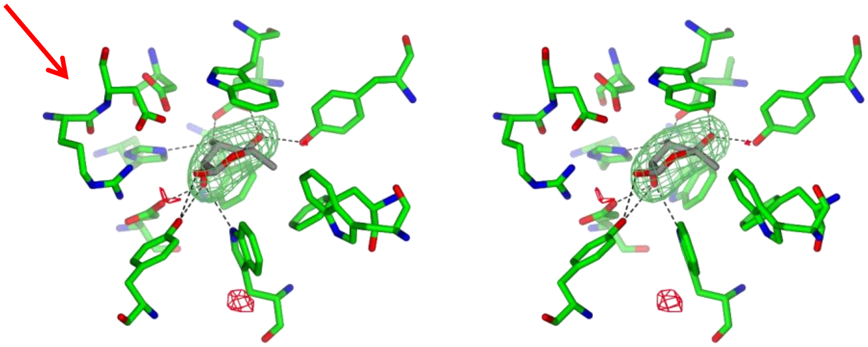

Supporting Information Fig. S3

Stereoviews of the rhamnose binding site in the same orientation as in figure 1b. The rhamnose is shown with light grey carbons and all the residues within 4 Å of the rhamnose are shown with green carbons. A red arrow indicates the position of the non-proline *cis*-peptide bond.

A: A $2F_{obs}$-F_{calc} electron density map (2.7 Å resolution, contoured at 1.0 σ) superposed on the final coordinates. Clear density can be seen for the rhamnose, all the surrounding residues including the non-proline *cis*-peptide bond. B: An omit electron density map for rhamnose (2.7 Å resolution, contoured at ±4.0 σ) and superposed on the final coordinates

(where green mesh indicates positive density and red mesh indicates negative density). Clear positive density can be seen for the rhamnose, and no difference density peaks are associated with the non-proline *cis*-peptide bond.

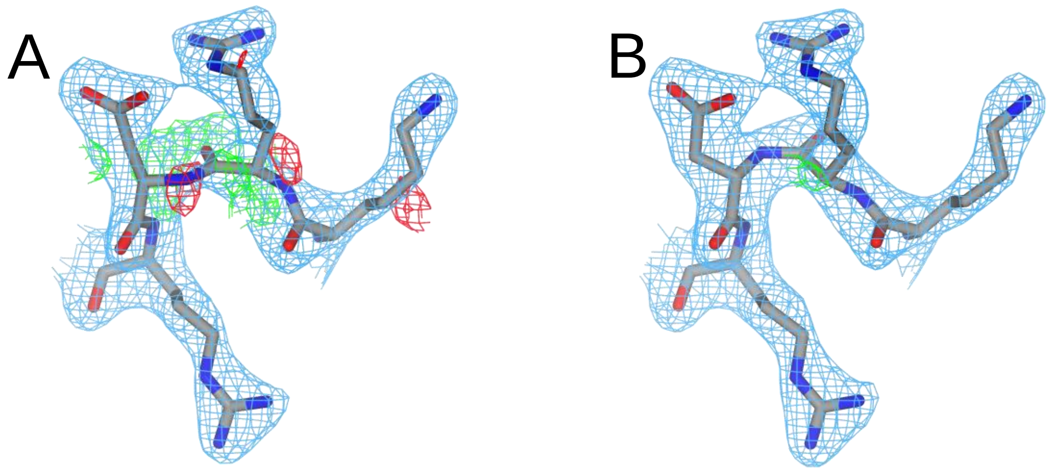

Supporting Information Fig. S4

Evidence for a non-proline *cis*-peptide bond in BT1001.

A: The data and coordinates for BT1001 (PDB entry 3CIH) were downloaded from the Electron Density Server (http://eds.bmc.uu.se/eds/). A large amount of difference density was associated with the peptide bond, modelled as *trans*, preceding the putative catalytic Asp. B: The same peptide bond re-modelled and re-refined as *cis*.

For both A and B, the blue mesh shows $2F_{obs}$-F_{calc} electron density (2.33 Å resolution, contoured at 1.0 σ) and the green and red meshes indicate positive and negative difference density, respectively (2.33 Å resolution, contoured at ±3.0 σ).

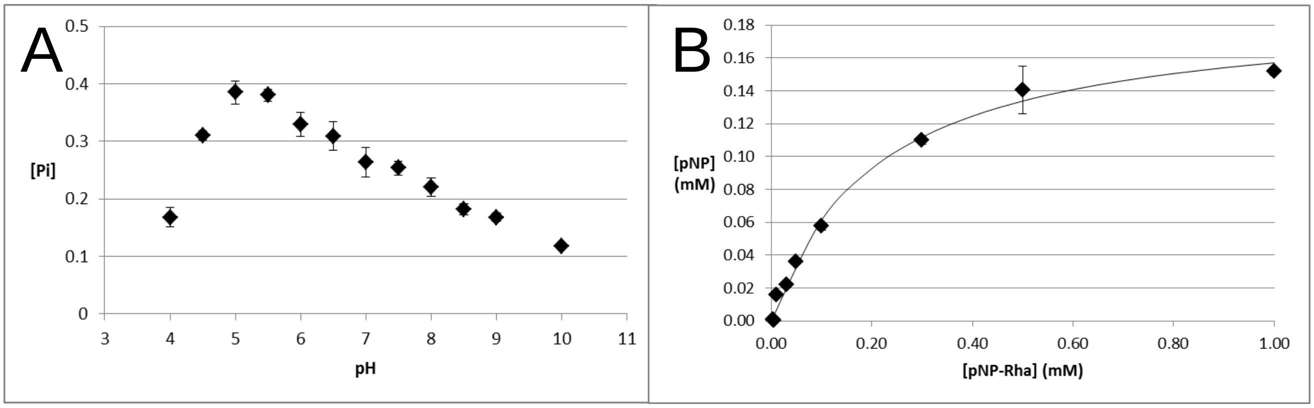

Supporting Information Fig. S5

A: pH optimum assay for KoRha. Assays were performed at a range of pH values (4-10) using a mixed buffer system (20 mM acetate, 20 mM MES, 20 mM Tris), measuring the hydrolysis of pNP-Rha (5 mM) by KoRha (21 µg/mL) in 1 hour at 22 °C. B: Kinetic activity assay for KoRha. Assays were performed at a range of pNP-Rha concentrations (0-1 mM) using KoRha (20 µg/mL) in assay buffer (20 mM MES, pH 5.0) in 10 mins at 22 °C. K_M = 0.21 ± 0.02 mM and V_{max} = 0.95 ± 0.003 µmol mg^{-1} min^{-1}. This is equivalent to a kcat of 0.98 s^{-1}.

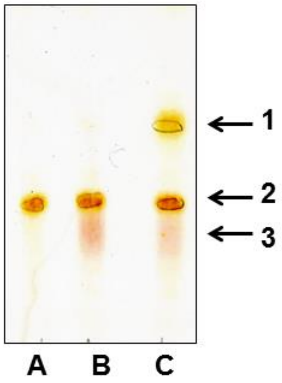

Supporting Information Fig. S6

Hydrolysis of rutin by KoRha. All assays were performed in MES buffer (20 mM, pH 5.0) containing MeOH (20%), separated by TLC and charred. A. Rutin (10 mM; **2**). B. Rutin (10 mM; **2**) plus rhamnose (10 mM; **3**). C. Rutin (10 mM) plus KoRha (100 µg/mL) showing three spots, corresponding to rutin (**2**), rhamnose (**3**) and arhamnosyl rutin (**1**).
